# Food Insecurity during Pregnancy in a Maternal–Infant Cohort in Brazilian Western Amazon

**DOI:** 10.3390/nu12061578

**Published:** 2020-05-28

**Authors:** Alanderson A. Ramalho, Cibely M. Holanda, Fernanda A. Martins, Bárbara T.C. Rodrigues, Débora M. Aguiar, Andréia M. Andrade, Rosalina J. Koifman

**Affiliations:** 1 Center for Health Sciences and Sports, Federal University of Acre, Rodovia BR 364, Km 04, Rio Branco AC 69920-900, Brazil; cibelymachado0410@gmail.com (C.M.H.); nutricionistafernanda@hotmail.com (F.A.M.); babi_cameli@hotmail.com (B.T.C.R.); debora_melo__@hotmail.com (D.M.A.); amasmsbg@hotmail.com (A.M.A.); 2 Department of Epidemiological and Quantitative Methods in Health, National School of Public Health Sergio Arouca, Oswaldo Cruz Foundation, Rua Leopoldo Bulhões 1480, Rio de Janeiro RJ 21041-210, Brazil; rosalina.koifman@hotmail.com

**Keywords:** food and nutrition security, pregnant women, pregnancy, maternal nutrition, nutrition surveys

## Abstract

The aim of this study was to determine the prevalence and analyze the factors associated with food insecurity during gestation in a maternal–infant cohort in Brazilian Western Amazon. A population-based cross-sectional study was conducted with parturients from a maternal–infant cohort in Rio Branco, located in the Western Brazilian Amazon. The dependent variable food insecurity (FI) was obtained through the Brazilian Scale of Food Insecurity, and associated factors were identified through multiple logistic regression. The prevalence of FI in pregnancy was of 34.8%. Regarding severity, the prevalence of mild food insecurity was 24.6%, moderate food insecurity was 4.8%, and severe food insecurity was 5.4%. The factors directly associated with FI were the presence of open sewage in the peridomestic environment; belonging to the lower economic classes; being an income transfer program beneficiary, while the factors inversely associated with FI were schooling equal to or greater than 8 years; having a partner; primigestation; and regular consumption of fruits and vegetables during pregnancy. These findings reinforce the need for the ratification of actions aimed at the domestic economy in the income transfer programs and the development of actions of food and nutritional education in the gestational period.

## 1. Introduction

Food security is the fulfillment of the right to regular and permanent access to safe, nutritious, and sufficient food to meet dietary needs and food preferences in order to lead an active and healthy life [1].

With such a comprehensive and integrated concept, the determination of food security demands indicators able to synthesize complex phenomena, such as food consumption, anthropometric status, and food availability, among other social and psychological factors [2]. In the 1980s and 1990s, different forms of evaluation were studied in the United States, resulting in the most applied psychometric scale in current research, the U.S. Household Food Security Survey Measure (HFSSM) [3,4]. The adaptation and validation of this instrument in Brazil resulted in the EBIA—Escala Brasileira de Insegurança Alimentar, or BFIS—Brazilian Food Insecurity Scale [5].

According to the report prepared by the United Nations Food and Agriculture Organization [6], the prevalence of food insecurity (FI) worldwide has improved, reducing from 18.6% in 1990–1992 to 10.9% in 2014–2016. In Latin America and the Caribbean, rates decreased from 14.7% to 5.5% during the same period. According to the Brazilian National Household Sample Survey (PNAD), the prevalence of FI in Brazilian households in 2013 was of 22.6% (20.5% in the urban area and 35.3% in the rural area). In the Northern region, FI was reported as 36.1%, and in the state of Acre, 31.2% [7].

FI is mainly determined by poverty and social inequalities, and studies that analyze factors associated with food insecurity are decisive for preventive and health promoting programs and public policies [2,8].

FI repercussions can be observed mainly among the most vulnerable groups. Infant mortality, impairment of physical and mental development, low birth weight, maternal mortality, increased school dropout, and reduced school performance are related to lack of healthy and quality food as a consequence of precarious access to income and goods and services [2,4,6]. Several studies point to a direct relationship between FI and decreased nutritional status in children [9,10,11,12,13].

Although studies on FI during the gestational period are available, population-based studies have been published only for the United States [14,15]. In Brazil, five studies analyzing FI in pregnant women were identified, with estimated prevalence ranging from 28.2% to 71.6%, but none was population-based [16,17,18,19,20] or carried out in the Brazilian Amazon. The Brazilian Amazon is divided into an Eastern and Western region. The Western Brazilian Amazon is made up of the states of Amazonas, Acre, Rondônia, and Roraima. This region holds 3.27% of the Brazilian population, 25.67% of the country’s land area [21] and comprises approximately 57% [22] of the Amazonian forests. This region comprises the greatest miscegenation and presence of indigenous people in the country and, despite its biodiversity, most of the food consumed by the population originates from other regions and is one of the Brazilian regions displaying the greatest socioeconomic inequalities [21]. In this context, the aim of the present study was to determine the prevalence and analyze the factors associated with FI during gestation in a maternal–infant cohort in Rio Branco, Acre, Brazilian Western Amazon.

## 2. Materials and Methods

### 2.1. Design and Study Population

This was a cross-sectional population-based study developed using a maternal–infant cohort in Rio Branco, Acre, Brazilian Western Amazon. The capital of the state of Acre concentrates 47.32% of the total population of the state, with 89.42% of the population located in the urban zone.

A total of 9638 children were born alive in 2015 in Rio Branco, and 27.7% of the mothers lived in another municipality. Of the 6965 live births whose mothers resided in Rio Branco, 99.89% of births took place in the only two maternity hospitals. Only 0.11% occurred in an out-of-hospital environment.

For the determination of the minimum sample size, an expected prevalence of 50% was adopted, with precision set for a sampling error of 0.05, at 95% confidence level, 80% power, and an odds ratio of 2. In these conditions, the minimum sample size was of 964 parturients. To account for non-response effects, 10% was added, resulting in an estimated sample of 1060 parturients.

Intrahospital delivery parturients in Rio Branco, Acre, who lived in the urban area and were hospitalized for delivery between April and June 2015 were included in this study. Parturients from multiple pregnancies were excluded. During the data collection period, 1205 women met the inclusion criteria, but 11 were excluded due to twin pregnancy.

### 2.2. Data Collection and Variables

Data collection took place in the only two maternity hospitals in Rio Branco, by means of a copy of hospital charts, a pregnancy card, and an interview using a semi-structured instrument to obtain socioeconomic, demographic, maternal habits, prenatal care, and delivery information. The data collection instrument was pre-tested and applied by health science academics. The interviews were carried out inside the maternity wards, with mothers approached about 12 h after delivery. Research assistants worked every day under a job rotation system to cover every day, full time. The interviewers were trained to obtain standardization and uniformity concerning data collection procedures.

The dependent food insecurity variable was obtained through the Brazilian Food Insecurity Scale (EBIA), nationally validated, comprising 15 structured questions in increasing order of seriousness, starting with issues related to concerns about the possibility of food shortage and the quality and amount of food in the family and ending with specific questions about lack of food for one or more days [5,23]. The development of this scale was based on the HFSSM (Household Food Security Survey Module) instrument, widely applied in other countries [24,25]. In 2010, the Technical Workshop for EBIA Analysis revised the scale, which now comprises 14 items. This was the version used in the present study, and each affirmative answer represented 1 point, with total scores ranging from 0 to 14 points [23].

In our study, questions were asked emphasizing the gestational period so that the reference was understood between the period of the pregnancy discovery and the last gestational weeks. The same questions from the EBIA validated in the study performed by Segall-Corrêa et al. [23] were applied herein. According to the EBIA scores, the women who scored 0 points were classified as experiencing a situation of food security during pregnancy; mild food insecurity (1 to 4 points), moderate food insecurity (5 to 9 points), and severe food insecurity (10 to 14 points) were also determined [23].

The independent variables were composed of socioeconomic and demographic conditions, prenatal care and gestational habits, hospital care, and newborn characteristics. The age variable was obtained by a direct and open question about age in years and confirmed by the difference between the date of the interview and the date of birth collected from personal documents at the time of the interview. Three categories were used (<20; 20–34; ≥35). Skin color was mentioned by the woman during the interview and corrected by the interviewer’s perception, under the following categories: white, black, yellow (Asian), brown (brunette/mulatto), red (indigenous), and others. As in Brazil, especially in the Western Brazilian Amazon, it is difficult to distinguish these categories precisely, we chose to present them as white and not white. Family income was transformed into minimum wages (MW) considering the minimum wage salary in 2015 (R$ 788.00) and presented as less than 1.5 MW and equal or higher than 1.5 MW. The socioeconomic class variable was defined by the Brazilian Association of Research Companies criteria of 2014 (Brazilian Economic Classification Criteria, http://www.abep.org/criterio-brasil, accessed on 15 October 2015). This criterion uses the presence of some consumer goods and the education level of the head of the family to classify the economic situation into A, B, C, D, and E. For the data analysis, these criteria were grouped into high (A and B) and low (C, D, and E) classes, due to the low frequency in classes A and E and the similarities of classes C and D. The frequency of food consumption was obtained by the regular consumption variable (5 times or more in the week) of fruits and vegetables, beans, meat, chicken, and milk. The variable type of service at delivery was dichotomized as public and private. The newborn characteristics were composed of the following variables: gender, low birth weight, and prematurity. The cutoff point for the definition of low birth weight was a birth weight below 2500 g and for preterm birth was gestational age less than 37 weeks.

### 2.3. Statistical Methods

The data were analyzed using the R software, version 3.3 (The R Foundation for Statistical Computing). The prevalence of the outcome was calculated with the respective confidence interval. To analyze the factors associated with the outcome of this study, a simple logistic regression was initially performed, selecting the independent variables that presented associations in other studies, in addition to those with *p* value below 0.20 for subsequent application in the multiple models. Crude odds ratios (ORcrude) and their respective 95% confidence intervals were presented. The next step involved the multiple logistic regression. In this step, the variables reported in the literature were maintained in the final model, which modified the general adjustment of the model and the magnitude of association of the ORs with their respective confidence intervals by up to 10%, in addition to variables with a *p*-value less than 0.05. After selecting the most parsimonious model, the adjusted odds ratios (ORadjust) and their respective confidence intervals were presented.

### 2.4. Ethics

The study was conducted in accordance with the Declaration of Helsinki, and the protocol was approved by the Ethics Committee of the Federal University of Acre (1.074.982) and the National School of Public Health (1.677.226). The researchers received authorization from the two institutions in which the data collection was performed. Informed written consent was obtained from all interviewees. For those under 18 years of age, written consent from the parents/guardians was also obtained. All interviewees were guaranteed the right of non-participation in the study, as well as confidentiality concerning the collected information.

## 3. Results

From the total 1194 participants, 18.9% were 13–19 years old, 32.5% were between 20 and 24 years old, 39.2% were between 25 and 34 years old, and 9.4% were 35 years of age or older (mean = 25.12, SD = 6.70). Of the total number of women, 10.6% declared that they were white and 37.3% declared that they had an income of less than 1.5 minimum wages. Regarding schooling, 6.5% studied up to elementary school 1, 19.4% up to elementary school 2, 51.4% up to high school, and 22.6% up to higher education.

The prevalence of FI during pregnancy was of 34.8% (95% CI = 32.2–37.5%). Regarding FI severity, the prevalence of mild FI was of 24.6% (95% CI = 22.3–27.0%), moderate prevalence was of 4.8% (95% CI = 3.6–6.0%) and severe prevalence was of 5.4% (95% CI = 4.3–6.8%). Increased food insecurity is associated with more unfavorable socioeconomic and demographic conditions. The pregnant women classified into more severe FI situations presented lower education levels, lower family income, were governmental family grant program recipients, and had no partner. The frequency of FI severity was also higher in homes where women were the head of the household and declared themselves non-white; in households with five or more residents; who had a resident below the age of 18 years old; homes with worse basic sanitation conditions, such as the presence of open sewage in the peridomestic environment, and the absence of a bathroom with toilet plumbing (Table 1 and Table 2).

Table 3 displays the FI distribution prevalence in gestation according to prenatal care and gestational habits. FI prevalence was higher and associated with no primigestation, a greater number of live children, and prenatal care in the public health system. It was also associated with smoking and drinking alcohol during pregnancy, diabetes in gestation, and non-regular consumption of red meat, milk, fruits, and vegetables during the gestational period, as well as the regular consumption of soft drinks or artificial juices. FI was also more frequent in women that experienced a normal delivery and public care at delivery (Table 4).

In the multivariable model, the chance of FI in households with open-air sewage in the peridomestic environment was 1.64-fold higher than the chance of FI in homes with open-air sewage households in the vicinity. The chance of FI was also 99% higher in economic classes C, D, and E, and 65% higher among governmental family grant program recipients. FI occurrence in women with a partner was 0.56-fold higher than FI chances for women with no partner. An inverse association between FI and schooling equal to or greater than eight years of study, regular consumption of fruits and vegetables, and primigestation was also observed, with protection estimated at 34%, 37%, and 41%, respectively (Table 5).

## 4. Discussion

In this study, the prevalence of FI in pregnancy was 34.8%. The factors directly associated with FI were the presence of open sewage in the peridomestic environment, belonging to the lower economic classes, being an income transfer program beneficiary; while the factors inversely associated with FI were schooling equal to or greater than 8 years, having a partner, primigestation, and regular consumption of fruits and vegetables during pregnancy.

Since the insertion of the EBIA in the National Household Sample Survey (PNAD) in 2004, Brazil has determined the prevalence of household food security in national surveys [7]. When comparing data from the last national survey to FI estimates for pregnant women in the urban area of Rio Branco, the prevalence of pregnant women undergoing FI was higher than that estimated by PNAD in 2013 for the Brazilian urban population (20.5%), and for the state of Acre (31.2%), and lower than the North (36.1%) and Northeast (38.1%) regions [7].

When stratified by severity, the prevalence of mild FI for pregnant women in the present study was higher than the PNAD estimates for Acre, North, and Brazil (13.9%, 21.6%, and 13.7%, respectively), while moderate and severe FI for pregnant women in Rio Branco was lower when compared to PNAD for the state of Acre, respectively, moderate: 6.1% and severe: 11.2%, while the North Region presents moderate and severe FI at 7.7% and 6.7% [7]. However, the PNAD groups results from both rural and urban areas and data from the capital and from isolated municipalities in the interior of Acre, whose access is only by river or air. The municipalities with the highest prevalence of malnutrition in Brazil are located in these remote areas of the state, which corroborates the fact that the prevalence of moderate and severe FI in Acre is higher than that in areas displaying the same classification in the North region and in the entire country.

Two studies in the interior of the state of Acre evaluated food safety in children and estimated FI prevalence higher than those observed for pregnant women in Rio Branco. In 2010, Frazao et al. [26], when studying schoolchildren aged 7 to 9 in the urban area of Acrelândia, estimated FI prevalence at 54% (mild FI: 32.4% and moderate and severe FI: 21.6%). In an urban census of households with children under five in Assis Brasil, in 2011, Ramalho et al. determined that the prevalence of FI was of 40.6% (mild: 24.1%, moderate: 10.5%; severe: 6.0%) [12], although these results should be evaluated carefully, since, in addition to the population groups being distinct, the Municipal Human Development Index (IDHM) of the capital is also higher (Rio Branco: 0.73; Acrelândia: 0.60; Assis Brasil: 0.59).

Despite general national and international surveys, lack of information for specific population groups, such as pregnant women, is still noted. The five national studies, identified herein that estimated the frequency of food security of pregnant women in Brazilian municipalities are not population-based. When comparing the results of the present study with these references, the prevalence of FI for pregnant women in the Rio Branco urban area was lower than those estimated in studies conducted in João Pessoa-PB (59.0%), [17], Recife–PE (71.6%) [16], Maceió–AL (42.7%) [19], Queimados and Petrópolis–RJ (37.8%) [18], and higher than the study carried out in Santo Antônio de Jesus-BA (28.16%) [20], although, the study in Bahia applied the six-item United States Department of Agriculture (USDA) short-scale food safety measurement instrument, while the other studies used the 14- or 15-item EBIA.

In Colombia, a study carried out with pregnant women attended to in the urban area in the city of Cartagena estimated FI prevalence as 29.8% (mild: 23%, moderate: 6.3%, severe: 0.5%) [27]. In the United States, two studies with pregnant women participating in the National Health and Nutrition Examination Survey (NHANES) population survey from 1999 to 2006, and from 1999 to 2008, determined FI as 15.7% and 21%, respectively [14,15].

The main factors associated with FI in pregnant women in Rio Branco refer to unfavorable socioeconomic conditions, such as the presence of open sewage in the peridomestic environment, belonging to economic classes C, D, and E, receiving a government family income transfer and having lower schooling levels.

Other studies also observed the association between FI in pregnant women and lower socioeconomic conditions. In Recife, among the food insecure mothers attended by three Family Health Units located in districts II and III, the chance of not having their own income was three-fold higher than that of those who presented a monthly income [16]. In a retrospective cohort with pregnant women attending university hospitals and private obstetrics clinics in North Carolina, USA, a direct association between FI and poorer social class was observed (OR = 4.84, 95% CI: 2.37–8.75) [28]. A similar situation was observed in adolescent pregnant women who underwent prenatal care in the three institutions providing health services belonging to the “ESE Salud Pereira” in Pereira, Colombia [29].

According to the PNAD 2013, the lower the monthly income of the family, the higher the proportion of households with moderate or severe FI [7]. The PNAD also noted that the increase in the severity of food insecurity decreases with the proportion of households covered by the sewage collection network: 63.2% with food security, 44.2% in light FI, and 34.4% in severe FI [7]. This direct association between FI and lower economic class, participation in income transfer programs, and worse sanitary conditions was also observed in the present study for pregnant women in Rio Branco, as well as in other studies carried out with different population groups [8,12,30,31,32].

In Rio Branco, the lower level schooling of pregnant women was directly associated with FI. For Brazil, both urban and rural, the higher the educational level of the residents, the lower the prevalence of moderate or severe FI. In 2013, 13.7% of the residents with 1 to 3 years of schooling were in a situation of moderate or severe FI, while for those with 15 years or more in study the percentage was 1.2% [7]. Other national studies also corroborate this assertion [8,30,31,32,33].

For the pregnant women in Rio Branco, the regular consumption of fruits and vegetables was inversely associated with FI, similar to what was observed by Lobo and collaborators in 19- to 35-year-old female parturients from two public maternity hospitals in João Pessoa, who estimated the magnitude of association between FI and regular consumption of raw salad at 0.79 (95% CI: 0.68–0.93); the magnitude of association for other factors was as follows: Cooked vegetables: OR = 0.87 (95% CI: 0.76−0.99); fresh fruit or fruit salad: OR = 0.64 (95% CI: 0.48–0.84) [17]. This association was also observed in a study carried out with the adult population of Campinas [8].

According to data from the 2016 Surveillance Program on risk factors and protection for chronic diseases by telephone survey (Vigitel), the prevalence of regular consumption of fruits and vegetables for adult women in Rio Branco is 30.3% (95% CI: 27.1–33.6%), being the third Brazilian capital that consumes this food group less regularly [34]. The same survey indicates that the prevalence of overweight in adult women in Rio Branco is 55.8% (95% CI: 52.2–59.5%) and that of obese adult women is 22.8% (95% CI: 19.7–25.9%), classifying this city as the most obese capital in the country and suggesting an inverse association between obesity and regular consumption of fruits, vegetables and vegetables [34]. This association is consistent with the results of a review of 23 Brazilian studies on the association between fruit and vegetable intake and overweight in children, adolescents, and adults [35].

The association between overweight and FI has been previously observed in different population groups [6,36,37,38,39,40], although it has not yet been well established during gestation. The relationship found by this study among low consumption of fruits and vegetables and food insecurity in pregnancy, concomitant with information from the capital’s Vigitel program, indicating the female population with the highest prevalence of overweight and obesity in Brazil, raises the hypothesis of association between food insecurity and overweight during pregnancy, and studies are required to elucidate the relationship between food consumption, gestational weight gain, and food insecurity.

Regarding marital situation, the inverse association between food insecurity and having a partner observed in pregnant women in Rio Branco is probably related to the social support increased by the family of the partner. A population-based study with adults in a metropolitan area of Rio de Janeiro, observed that individuals with high social support rates presented a lower chance of moderate food insecurity (OR = 0.96, 95% CI: 0.94–0.99) and severe FI (OR = 0.96, 95% CI: 0.94–0.98) [41]. Another study carried out with pregnant women attended at the regional referral hospital in Gulu, Uganda, observed that the association between food insecurity and severity of depressive symptoms was moderated by social support, that is, it was stronger among women in the low social support category (adjusted beta: 0.91, 95% CI: 0.55–1.27) than among women in the high social support group (adjusted beta 0.53, 95% CI 0.28–0.78, *p* value adjusted for interaction = 0.026) [42]. Tsai et al. also observed similar results when studying food insufficiency, one of the aspects of food insecurity, in a cohort of population-based pregnant women in the periurban region of Cape Town, South Africa [43].

In this study, despite the existence of income transfer programs, regular consumption of fruits and vegetables is still relatively low and was associated with food insecurity in pregnant women. This result highlights the social inequalities experienced in remote regions such as the Western Brazilian Amazon [7,8,21]. Rio Branco, capital of Acre where this study was developed, is one of the Brazilian capitals with the lowest Human Development Index, which partially explains why the prevalence of food insecurity found in this study has been higher than other national findings [7,8,12,30,31,32] and the factors associated with this result, mainly of a socioeconomic nature.

This is the first population-based study on food insecurity during pregnancy conducted in the city of Rio Branco, Acre and in the Western Brazilian Amazon. Despite this, this study displays certain limitations regarding the cross-sectional design and for having used retrospective information, which may lead to memory bias. However, to minimize the memory bias, the interviewers were trained in such a way that the moment of discovering the pregnancy as a reference point was evident. In addition, the period of recall of these data was short and it is a remarkable event in a woman’s life.

## 5. Conclusions

The prevalence of food insecurity in gestation in Rio Branco was 34.7%, directly associated with the presence of open sewage in the peridomestic environment, belonging to lower socioeconomic classes, being beneficiaries of an income transfer program, and inversely associated with schooling equal to or greater than 8 years, having a partner, primigestation, and having regularly consumed fruits and vegetables during pregnancy.

These findings reinforce that promoting access to low-cost, nutritionally adequate, and safe foods (without compromising access to other items essential to life) is paramount for the promotion of food security.

In the Amazon context, it is essential to ratify actions aimed at the domestic economy in income transfer programs and to develop food and nutrition education actions during pregnancy. It would be opportune to take actions that encourage the consumption of regional foods that are not frequently consumed, encouraging the creation of vegetable gardens and family farming, strategies for the full use of food, and the generation of informal income through the sale of these foods, among others.

In addition, investments in actions that provide an increase in schooling in a timely manner, as well as policies that increase female insertion and permanence in the formal labor market may also influence the reduction of food insecurity within this vulnerable group.

## Figures and Tables

**Table 1:** Percentage distribution of socioeconomic, demographic, and health characteristics according to food insecurity (FI). Rio Branco, Acre, 2015.

Variable	Total	Food Security	Light Food Insecurity	Moderate Food Insecurity	Severe Food Insecurity	*p*-Value
		n (%)	n (%)	n (%)	n (%)	
**Type of paving of the residence street (*n* = 1193)**						0.261
Asphalt/cement/paving stone/brick	885 (74.2)	589 (66.6)	213 (24.1)	41 (4.6)	42 (4.7)	
Earth with other materials or just earth	308 (25.8)	189 (61.4)	81 (26.3)	16 (5.2)	22 (7.1)	
**Open sewage (*n* = 1186)**						0.001
No	920 (77.6)	626 (68.0)	203 (22.1)	45 (4.9)	46 (5.0)	
Yes	226 (22.4)	147 (55.3)	89 (33.5)	12 (4.5)	18 (6.8)	
**Bathroom with toilet plumbing (*n* = 1179)**						<0.001
No	209 (17.7)	107 (51.2)	62 (29.7)	17 (8.1)	23 (11.0)	
Yes	970 (82.3)	658 (67.8)	231 (23.8)	39 (4.0)	42 (4.3)	
**Age (*n* = 1194)**						0.365
<20	311 (26.0)	203 (65.3)	83 (26.7)	14 (4.5)	11 (3.5)	
20 to 34	762 (63.8)	502 (65.9)	180 (23.6)	37 (4.9)	43 (5.6)	
≥35	121 (10.1)	73 (60.3)	31 (25.6)	6 (5.0)	11 (9.1)	
**Skin color (*n* = 1193)**						0.03
White	126 (10.6)	95 (75.4)	26 (20.6)	3 (2.4)	2 (1.6)	
Not white	1067 (89.4)	682 (63.9)	268 (25.1)	54 (5.1)	63 (5.9)	
**Schooling (*n* = 1194)**						<0.001
Up to elementary school 1	78 (6.5)	38 (48.7)	16 (20.5)	9 (11.5)	15 (19.2)	
Elementary school 2	232 (19.4)	119 (51.3)	71 (30.6)	19 (8.2)	23 (9.9)	
High school	614 (51.4)	402 (65.5)	164 (26.7)	23 (3.7)	25 (4.1)	
Higher education	270 (22.6)	219 (81.1)	43 (15.9)	6 (2.2)	2 (0.7)	
**Marital status (*n* = 1193)**						0.013
No partner	191 (16.0)	107 (56.0)	56 (29.3)	11 (5.8)	17 (8.9)	
With partner	1002 (84.0)	671 (67.0)	238 (23.8)	46 (4.6)	47 (4.7)	
**Head of the family (*n* = 1194)**						0.013
The interviewee	158 (13.2)	91 (57.6)	40 (25.3)	11 (7.0)	16 (10.1)	
Partner or other	1036 (86.8)	687 (66.3)	254 (24.5)	46 (4.4)	49 (4.7)	
**Head of family schooling (*n* = 1157)**						<0.001
No school	54 (4.7)	30 (55.6)	14 (25.9)	3 (5.6)	7 (13.0)	
Up to elementary school 1	132 (11.4)	69 (52.3)	34 (25.8)	10 (7.6)	19 (14.4)	
Elementary school 2	203 (17.5)	118 (58.1)	52 (25.6)	18 (8.9)	15 (7.4)	
High school	525 (45.4)	336 (64.0)	148 (28.2)	19 (3.6)	22 (4.2)	
Higher education	243 (21.0)	196 (80.7)	40 (16.5)	5 (2.1)	2 (0.8)	
**Number of residents per household (*n* = 1194)**						0.004
1 or 2	334 (28.0)	230 (68.9)	80 (24.0)	10 (3.0)	14 (4.2)	
3 to 5	549 (46.0)	361 (65.8)	141 (25.7)	22 (4.0)	25 (4.6)	
5 or more	311 (26.0)	187 (60.1)	73 (23.5)	25 (8.0)	26 (8.4)	
**Any residents under the age of 18 (*n* = 1194)**						<0.001
No	379 (31.7)	278 (73.4)	78 (20.6)	11 (2.9)	12 (3.2)	
Yes	815 (68.3)	500 (61.3)	216 (26.5)	46 (5.6)	53 (6.5)	
**Any residents under the age of 15 (*n* = 1194)**						<0.001
No	496 (41.5)	359 (72.4)	106 (21.4)	17 (3.4)	14 (2.8)	
Yes	698 (58.5)	419 (60.0)	188 (26.9)	40 (5.7)	51 (7.3)	
**Family income in minimum salaries (*n* = 1020)**						<0.001
Less than 1	160 (15.7)	72 (45.0)	50 (31.3)	16 (10.0)	22 (13.8)	
1 to 2.9	578 (56.7)	348 (60.4)	166 (28.7)	30 (5.2)	34 (5.9)	
3 or more	282 (27.6)	228 (80.9)	49 (17.4)	3 (1.1)	2 (0.7)	
**Government program (*n* = 1133)**						<0.001
No	917 (80.9)	633 (69.0)	213 (23.2)	35 (3.8)	36 (3.9)	
Yes	216 (19.1)	98 (45.4)	70 (32.4)	20 (9.3)	28 (13.0)	
**Socioeconomic class (*n* = 1181)**						<0.001
High (A and B)	242 (20.5)	198 (81.8)	37 (15.3)	6 (2.5)	1 (0.4)	
Low (C, D, and E)	935 (79.5)	569 (60.6)	256 (27.3)	50 (5.3)	64 (6.8)	
**Paid work (*n* = 1141)**						< 0.001
No	732 (64.2)	446 (60.9)	196 (26.8)	43 (5.9)	47 (6.4)	
Yes	409 (35.8)	301 (73.6)	89 (21.8)	9 (2.2)	10 (2.4)	
**Primigestation (*n* = 1193)**						<0.001
No	723 (60.6)	427 (59.1)	201 (27.8)	44 (6.1)	51 (7.1)	
Yes	470 (39.4)	350 (74.5)	93 (19.8)	13 (2.8)	14 (3.0)	
**Type of prenatal care (*n* = 1158)**						<0.001
Public	982 (84.8)	606 (61.7)	266 (27.1)	47 (4.8)	63 (6.4)	
Private	176 (15.2)	145 (82.4)	25 (14.2)	6 (3.4)	0 (0.0)	
**Hypertension during pregnancy (*n* = 1192)**						0.542
No	1008 (84.6)	656 (65.1)	245 (24.3)	52 (5.2)	55 (5.5)	
Yes	184 (15.4)	121 (65.8)	48 (26.1)	5 (2.7)	10 (5.4)	
**Anemia during pregnancy (*n* = 992)**						0.512
No	855 (86.2)	554 (64.8)	212 (24.8)	41 (4.8)	48 (5.6)	
Yes	137 (13.8)	97 (70.8)	30 (21.9)	4 (2.9)	6 (4.4)	
**Diabetes during pregnancy (*n* = 1186)**						0.277
No	1086 (91.6)	717 (66.0)	262 (24.1)	49 (4.5)	58 (5.3)	
Yes	100 (8.4)	58 (58.0)	28 (28.0)	8 (8.0)	6 (6.0)	
**Frequency of consumption of fruits and vegetables during pregnancy (*n* = 1187)**						<0.001
Less than 5 times a week	925 (77.9)	578 (62.5)	234 (25.3)	53 (5.7)	60 (6.5)	
5 times or more	262 (22.1)	196 (74.8)	58 (22.1)	4 (1.5)	4 (1.5)	
**Type of childbirth service (*n* = 1190)**						<0.001
Public	1064 (89.4)	670 (63.0)	277 (26.0)	53 (5.0)	64 (6.0)	
Private	126 (10.6)	104 (82.5)	17 (13.5)	4 (3.2)	1 (0.8)	

**Table 2:** Food insecurity during gestation according to socioeconomic and demographic characteristics. Rio Branco, Acre, 2015.

	Food Insecurity		Food Security			
Variable	*n*	%	*n*	%	ORcrude	CI 95%
**Type of paving of the domicile street (*n* = 1193)**						
Asphalt/cement/paving stone/brick	296	71.3	589	75.7	1	
Earth with other materials or just earth	119	28.7	189	24.3	1.25	0.96–1.64
**Open sewage (*n* = 1186)**						
No	294	71.2	626	81.0	1	
Yes	119	28.8	147	19.0	1.72	1.30–2.28
**Bathroom with toilet plumbing (*n* = 1179)**						
No	102	24.6	107	14.0	1	
Yes	312	75.4	658	86.0	0.50	0.37–0.67
**Age (*n* = 1194)**						
13 to 19	75	18.0	147	18.9	1	
20 to 24	135	32.5	253	32.5	1.05	0.74–1.48
25 to 34	156	37.5	305	39.2	1.01	0.71–1.41
35 or more	50	12.0	73	9.4	1.34	0.85–2.12
**Skin color (*n* = 1193)**						
White	31	7.5	95	12.2	1	
Not white	385	92.5	682	87.8	1.73	1.13–2.64
**Schooling (*n* = 1194)**						
Up to elementary school 1	40	9.6	38	4.9	1	
Elementary school 2	113	27.2	119	15.3	0.90	0.54–1.51
High school	212	51.0	402	51.7	0.50	0.31–0.80
Higher education	51	12.3	219	28.1	0.22	0.13–0.38
**Marital status (*n* = 1193)**						
No partner	84	20.2	107	13.8	1	
With partner	331	79.8	671	86.2	0.63	0.46–0.86
**Head of the family (*n* = 1194)**						
The interviewee	67	16.1	91	11.7	1	
Partner or other	349	83.9	687	88.3	0.69	0.49–0.97
**Head of family schooling (*n* = 1157)**						
Up to elementary school 1	87	21.0	99	13.2	1	
Elementary school 2	85	20.8	118	15.8	0.82	0.55–1.22
High school	189	46.3	336	44.9	0.64	0.46–0.90
Higher education	47	11.5	196	26.2	0.27	0.18–0.42
**Number of residents per household (*n* = 1194)**						
1 or 2	104	25.0	230	29.6	1	
3 to 5	188	45.2	361	46.4	1.15	0.86–1.54
5 or more	124	29.8	187	24.0	1.47	1.06–2.03
**Any residents under the age of 18 (*n* = 1194)**						
No	101	24.3	278	35.7	1	
Yes	315	75.7	500	64.3	1.73	1.33–2.27
**Any residents under the age of 15 (*n* = 1194)**						
No	137	32.9	359	46.1	1	
Yes	279	67.1	419	53.9	1.74	1.36–2.24
**Family income in minimum salaries (*n* = 1020)**						
Less than 1	88	23.7	72	11.1	1	
1 to 2.9	230	61.8	348	53.7	0.54	0.38–0.77
3 or more	54	14.5	228	35.2	0.19	0.13–0.30
**Government program (*n* = 1133)**						
No	284	70.6	633	86.6	1	
Yes	118	29.4	98	13.4	2.68	1.98–3.63
**Socioeconomic class (*n* = 1181)**						
High (A and B)	44	10.6	198	25.8	1	
Low (C, D, and E)	370	89.4	569	74.2	2.93	2.06–4.16

**Table 3:** Food insecurity in gestation according to prenatal care and gestational habits. Rio Branco, Acre, 2015.

	Food Insecurity		Food Security			
Variable	*n*	%	*n*	%	ORcrude	CI 95%
**First pregnancy (*n* = 1193)**						
No	296	71.2	427	55.0	1	
Yes	120	28.8	350	45.0	0.49	0.38–0.64
**Number of prenatal consultations (*n* = 1170)**						
None	3	0.7	6	0.8	1	
1 to 6	131	32.2	194	25.4	1.35	0.33–5.50
6 to 8	191	46.9	348	45.6	1.1	0.27–4.44
8 or more	82	20.1	215	28.2	0.76	0.19–3.12
**Type of prenatal care (*n* = 1158)**						
Public	376	92.4	606	80.7	1	
Private	31	7.6	145	19.3	0.34	0.23–0.52
**Number of living children (*n* = 1191)**						
None	117	28.3	351	45.2	1	
1 or 2	126	30.4	243	31.3	1.56	1.15–2.10
3 or more	171	41.3	183	23.6	2.80	2.09–3.77
**Smoked during pregnancy (*n* = 1194)**						
No	358	86.1	721	92.7	1	
Yes	58	13.9	57	7.3	2.05	1.39–3.02
**Alcoholic beverage intake during pregnancy (*n* = 1184)**						
No	345	83.9	692	89.5	1	
Yes	66	16.1	81	10.5	1.63	1.15–2.32
**Gestational weight gain reported by a professional (*n* = 1178)**						
they did not say anything	41	10.0	44	5.7	1	
said that the weight gain was adequate	191	46.6	409	53.3	0.5	0.32–0.79
said they were gaining a lot of weight	115	28.0	233	30.3	0.53	0.33–0.86
said they were gaining little weight	63	15.4	82	10.7	0.82	0.48–1.41
**Hypertension during pregnancy (*n* = 1192)**						
No	352	84.8	656	84.4	1	
Yes	63	15.2	121	15.6	0.97	0.70–1.35
**Anemia during pregnancy (*n* = 992)**						
No	301	88.3	554	85.1	1	
Yes	40	11.7	97	14.9	0.76	0.51–1.13
**Diabetes during pregnancy (*n* = 1186)**						
No	369	89.8	717	92.5	1	
Yes	42	10.2	58	7.5	1.41	0.93–2.13
**Urinary tract infection during pregnancy (*n* = 1191)**						
No	173	41.9	352	45.2	1	
Yes	240	58.1	426	54.8	1.15	0.90–1.46
**Syphilis during pregnancy (*n* = 1191)**						
No	399	95.9	752	97.0	1	
Yes	17	4.1	23	3.0	1.39	0.74–2.64
**Hospitalization during pregnancy (*n* = 1156)**						
No	332	82.4	651	86.5	1	
Yes	71	17.6	102	13.5	1.36	0.98–1.90
**Frequency of bean consumption during pregnancy (*n* = 1193)**						
Less than 5 times a week	172	41.3	291	37.5	1	
5 times or more	244	58.7	486	62.5	0.85	0.67–1.08
**Frequency of fruit and vegetable consumption during pregnancy (*n* = 1187)**						
Less than 5 times a week	347	84.0	578	74.7	1	
5 times or more	66	16.0	196	25.3	0.56	0.41–0.76
**Frequency of red meat consumption during pregnancy (*n* = 1192)**						
Less than 5 times a week	253	60.8	455	58.6	1	
5 times or more	163	39.2	321	41.4	0.91	0.72–1.16
**Frequency of chicken consumption during pregnancy (*n* = 1191)**						
Less than 5 times a week	355	85.5	687	88.5	1	
5 times or more	60	14.5	89	11.5	1.30	0.92–1.85
**Frequency of consumption of natural fruit juice during pregnancy (*n* = 1191)**						
Less than 5 times a week	303	72.8	475	61.3		1
5 times or more	113	27.2	300	38.7	0.59	0.46–0.77
**Frequency of the consumption of soft drinks and artificial juice during pregnancy (*n* = 1189)**						
Less than 5 times a week	273	65.9	580	74.8		1
5 times or more	141	34.1	195	25.2	1.54	1.18–1.99
**Frequency of milk consumption during pregnancy (*n* = 1186)**						
Less than 5 times a week	141	34.1	163	21.1	1	
5 times or more	272	65.9	610	78.9	0.52	0.39–0.67
**Consumption of meat or chicken with excess fat (*n* = 1175)**						
No	272	66.5	535	69.8	1	
Yes	137	33.5	231	30.2	1.17	0.90–1.51

**Table 4:** Food insecurity during pregnancy according to delivery and weight of the newborn. Rio Branco, 2015.

	Food Insecurity		Food Security			
Variable	*n*	%	*n*	%	ORcrude	CI 95%
**Type of delivery (*n* = 1192)**						
Normal	237	57.1	383	49.3	1	
Caesarean	178	42.9	394	50.7	0.73	0.57–0.93
**Delivery unit (*n* = 1194)**						
Unit A	277	66.6	476	61.2	1	
Unit B	139	33.4	302	38.8	0.79	0.62–1.02
**Type of childbirth service (*n* = 1190)**						
Public	394	94.7	670	86.6	1	
Private	22	5.3	104	13.4	0.36	0.22–0.58
**Low weight at birth (*n* = 1188)**						
No	379	91.8	708	91.4	1	
Yes	34	8.2	67	8.6	0.95	0.62–1.46
**Preterm (*n* = 1184)**						
No	365	89.2	692	89.3	1	
Yes	44	10.8	83	10.7	1.01	0.69–1.48

**Table 5:** Factors associated with food insecurity during pregnancy in a cohort in Rio Branco, AC, 2015.

Variable	ORcrude	CI 95%	ORadjust	CI 95%
**Open sewage**				
No	1		1	
Yes	1.72	1.30–2.28	1.64	1.21–2.22
**Socioeconomic class**				
High (A and B)	1		1	
Low (C, D, and E)	2.93	2.06–4.16	1.99	1.35–2.94
**Government program**				
No	1		1	
Yes	2.68	1.98–3.63	1.65	1.18–2.30
**Schooling**				
Up to 8 years	1		1	
8 years or more	0.43	0.33–0.57	0.66	0.49–0.90
**Marital status**				
No partner	1		1	
With partner	0.63	0.46–0.86	0.56	0.39–0.79
**Primigestation**				
No	1		1	
Yes	0.49	0.38–0.64	0.59	0.44–0.78
**Frequency of fruit and vegetable consumption during pregnancy**				
Less than 5 times a week	1		1	
5 times or more	0.56	0.41–0.76	0.63	0.45–0.88
